# Maternal Serum Screening Markers and Adverse Outcome: A New Perspective

**DOI:** 10.3390/jcm3030693

**Published:** 2014-07-03

**Authors:** David Krantz, Terrence Hallahan, David Janik, Jonathan Carmichael

**Affiliations:** PerkinElmer Labs/NTD, 80 Ruland Road, Suite 1, Melville, NY 11747, USA; E-Mails: terry.hallahan@perkinelmer.com (T.H.); david.janik@perkinelmer.com (D.J.); jon.carmichael@perkinelmer.com (J.C.)

**Keywords:** aneuploidy screening, preeclampsia, IUGR, preterm birth, fetal loss, placenta accreta, open neural tube defects

## Abstract

There have been a number of studies evaluating the association of aneuploidy serum markers with adverse pregnancy outcome. More recently, the development of potential treatments for these adverse outcomes as well as the introduction of cell-free fetal DNA (cffDNA) screening for aneuploidy necessitates a re-evaluation of the benefit of serum markers in the identification of adverse outcomes. Analysis of the literature indicates that the serum markers tend to perform better in identifying pregnancies at risk for the more severe but less frequent form of individual pregnancy complications rather than the more frequent but milder forms of the condition. As a result, studies which evaluate the association of biomarkers with a broad definition of a given condition may underestimate the ability of such markers to identify pregnancies that are destined to develop the more severe form of the condition. Consideration of general population screening using cffDNA solely must be weighed against the fact that traditional screening using serum markers enables detection of severe pregnancy complications, not detectable with cffDNA, of which many may be amenable to treatment options.

## 1. Introduction

Prenatal screening for birth defects was initially implemented using a single biochemical marker (alpha-fetoprotein) to identify a single condition (open neural tube defects, ONTDs) in the second trimester of pregnancy [1,2]. Over the course of the last 30 years, the field has evolved so that multiple ultrasound and biochemical markers across the first and second trimesters are used to identify patients at risk not only for ONTDs but also for Down syndrome and trisomy 18/13 [3,4,5,6,7,8,9,10,11]. In addition, there have been a number of reports [12,13,14,15,16,17,18] regarding the effectiveness of the serum markers to identify pregnancies at high risk for additional adverse perinatal outcomes leading to a number of reviews and consensus opinions [19,20,21,22,23,24]. The purpose of such reviews was to evaluate serum markers which were already being used in aneuploidy screening to see if there was any additional benefit in identifying other conditions beyond the primary outcomes being screened. These reviews focused on improving pregnancy management through the use of additional counseling and follow-up ultrasound examination since the effectiveness of treatments for these other conditions was not well-established.

**Figure 1:**
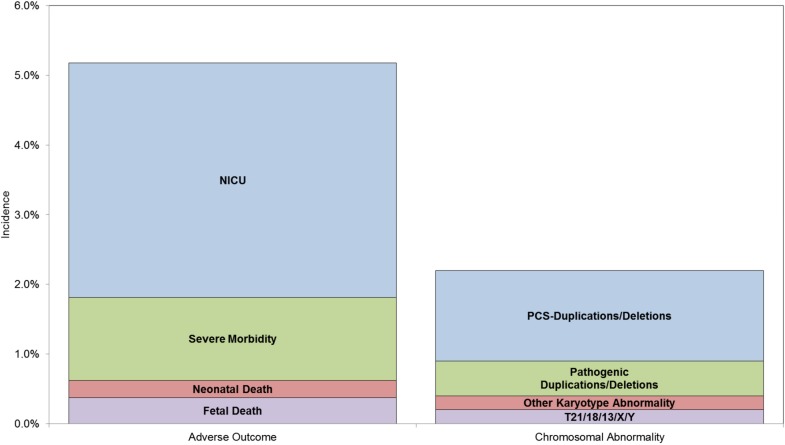
Comparison of incidence of adverse outcomes due to all causes with incidence of chromosomal abnormality. Data for incidence of adverse outcome from Lisonkova *et al.* [35]. Data for incidence of chromosomal abnormality from Wapner *et al.* [36]. NICU = Infant admitted to neonatal intensive care unit, PCS-Duplications/Deletions = Chromosome microdeletions and duplications with potential for clinical significance, T21/18/13/X/Y = trisomy 21, trisomy 18, trisomy 13 and sex chromosome abnormalities.

More recently, there have been new developments that need to be considered when evaluating aneuploidy screening markers for other adverse outcomes. Evaluation of cell-free fetal DNA (cffDNA) in maternal blood offers the opportunity to significantly improve the detection of Down syndrome while substantially reducing false positive rates [25,26,27,28]. This new technology can also be used to detect trisomy 18, 13 and sex chromosome abnormalities [28,29,30] albeit at somewhat lower detection rates than for trisomy 21. Based on the initial studies, the American Congress of Obstetricians and Gynecologists (ACOG) [31] concluded that cffDNA-based testing could be offered to pregnancies at high risk for aneuploidy including those with advanced maternal age. Before it can be determined whether or not cffDNA testing should be applied to a low risk population a number of factors need to be considered. First, the cost of the technology is significant and at present the cost of providing cffDNA testing to the entire population is substantially greater than that of current screening protocols even after factoring in the savings due to improved detection [32]. The cffDNA approach is highly focused on the specific genetic disorders tested for and therefore, at present cffDNA testing cannot detect the significant percentage of atypical abnormal karyotype results which are associated with abnormal l serum or nuchal translucency values [33]. Furthermore, cffDNA testing does not appear to be useful in the identification of other adverse perinatal outcomes which can lead to severe perinatal morbidity and mortality such as preeclampsia, preterm birth and small for gestational age neonates [34]. Indeed, the incidence of severe morbidity, mortality and NICU admission exceeds the incidence of the genetic disorders that can be identified by cffDNA testing (Figure 1). The ability to reduce the incidence of severe morbidity and mortality lies in the early identification and treatment of asymptomatic pregnancies.

Initial meta-analysis of 31 randomized trials indicated that aspirin had only a small beneficial effect on reducing the incidence of preeclampsia as a whole [37]. However, re-analysis of those studies by Bujold *et al.* [38] and Roberge *et al.* [39,40] showed that when aspirin was administered prior to 16 weeks the incidence of preeclampsia was reduced by 53%. Restricting the analysis to early-onset preeclampsia showed a reduction in incidence of approximately 90% [38]. In addition, the analysis showed that aspirin treatment prior to 16 weeks was effective in reducing fetal loss, fetal growth restriction, and preterm birth [38]. Other studies have reported on effective interventions for preterm birth such as cervical cerclage or progesterone [41,42,43,44,45]. In addition, fetal surgery is now a potential option in treating myelomeningocele [46,47,48]. Finally, early identification of placenta accreta can prepare the medical team for potential complications during delivery [49,50,51]. Below we review the association of adverse outcomes with respect to routine serum screening markers.

## 2. Preeclampsia

Several studies have evaluated first trimester free β human chorionic gonadotropin (free hCGβ) and pregnancy associated plasma protein A (PAPP-A) as markers for preeclampsia [17,52,53,54,55,56]. In general, these studies did not show an association of preeclampsia with free hCGβ but did show an association with low PAPP-A resulting in detection rates of 8%–15% at a false positive rate of 5%.

Morris *et al.* [23] performed a systematic meta-analysis of cohort studies evaluating second trimester markers and preeclampsia. There was significant variation among studies in the threshold used to identify patients at high-risk as well as significant variation in screening performance. The most effective thresholds were 2.0 multiples of the median (MoM) for alpha-fetoprotein (AFP) resulting in a positive likelihood ratio (LR) of 2.36 and a negative likelihood ratio of 0.96; 2.0 MoM for hCG resulting in a positive LR of 2.45 and a negative LR of 0.89; 0.5 MoM for unconjugated estriol (uE3) resulting in a positive LR of 1.50 and a negative LR of 0.99 and 2.79 MoM for inhibin (dimeric inhibin A) resulting in a positive LR of 19.5 and a negative LR of 0.30. Similarly, Kang *et al.* [57] found a significant association between inhibin and preeclampsia but not AFP and uE3. In a 3 marker protocol including PAPP-A, inhibin and hCG, the detection rate was 40% at a 5% false positive rate. The FASTER trial [18] also showed an association between preeclampsia and inhibin with a detection rate of 17% at a false positive rate of 3% but detection was not improved with additional markers.

Among preeclampsia pregnancies, approximately 70% of perinatal deaths and 60% of cases of severe neonatal morbidity occur in early onset (<34 weeks) preeclampsia even though these cases represent only about 10% of all preeclampsia cases [35]. As a result, a significant positive impact on perinatal morbidity and mortality can be achieved with effective screening programs for the early-onset form of the disease.

Olsen *et al.* [58] found that elevated levels of inhibin, hCG and AFP could each identify 22%–28% of early onset preeclampsia (<34 weeks) at an approximate 5% false positive rate and that the association of these markers with early onset preeclampsia was stronger than the association with late onset preeclampsia. Other studies indicate that PAPP-A may also perform better as a marker for early onset-preeclampsia rather than late-onset preeclampsia [59,60,61]. Kang *et al.* [57] found that inhibin was more strongly associated with early rather than late onset preeclampsia. Huang *et al.* [62] developed a multiple marker algorithm utilizing first trimester PAPP-A and second trimester AFP, hCG and uE3 to identify 18% of early onset (<32 weeks) preeclampsia. Inclusion of inhibin and maternal characteristics (such as previous history or family history of preeclampsia, parity and chronic hypertension) into such a protocol could potentially lead to significantly higher detection.

Recent data has indicated that a direct screen including maternal characteristics, PAPP-A, placental growth factor (PlGF), uterine artery doppler pulsatility index and mean arterial pressure can identify over 90% of early-onset preeclampsia pregnancies in the first trimester [63,64,65]. Coincidentally, PlGF along with AFP have been demonstrated to be effective in first trimester Down syndrome screening when combined with PAPP-A and free hCGβ [66,67,68]. Thus, an expanded Down syndrome screening protocol may lead to early identification of early-onset preeclampsia.

## 3. Intrauterine Growth Restriction

Until recently, the terminology used to describe intrauterine growth restriction (IUGR) has been inconsistent and confusing [69] and the term small for gestational age (SGA) has been used interchangeably with IUGR. An estimated fetal weight below the tenth percentile can alert the clinician to small fetal size but does not effectively differentiate between those fetuses who are small for pathological reasons and those that are constitutionally small but healthy. In an effort to differentiate between pathologically small and constitutionally small fetuses, the PORTO study [70] evaluated stricter criteria for the classification of IUGR. The authors found that those pregnancies more likely to have adverse pregnancy outcome or NICU admissions had estimated fetal weight <3rd percentile or abnormal umbilical artery (UA) Doppler compared to those pregnancies with normal UA Doppler and estimated fetal birth weight between the 3rd and 10th percentiles.

**Figure 2:**
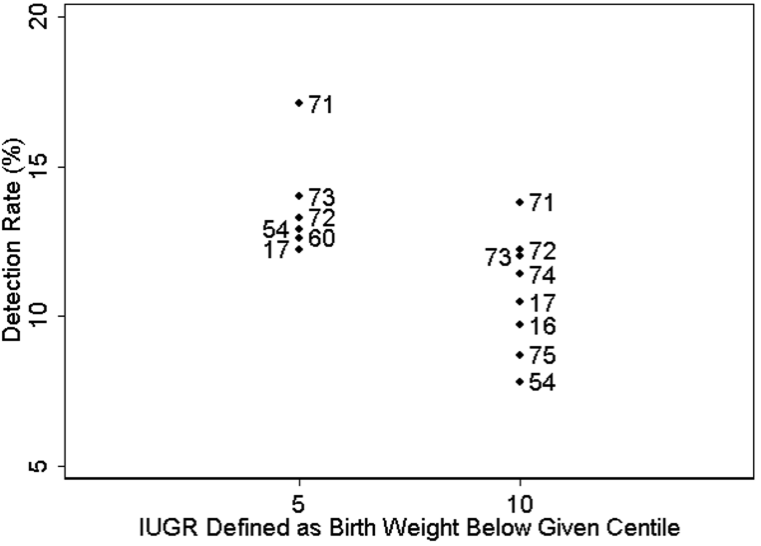
Detection Rate of IUGR using PAPP-A. The numbers indicate the associated references.

In general, studies of serum screening markers have used birth weight rather than estimated fetal weight to describe IUGR. There appears to be a tendency for extreme analyte values to be associated with more extreme low birth weight. D’antonio *et al.* [60], recently summarized the literature on the association of PAPP-A with IUGR. The detection rate of IUGR when it was defined as birth weight below the fifth percentile [17,54,60,71,72,73] is generally greater than when it was defined as birth weight below the tenth percentile [16,17,54,71,72,73,74,75] (Figure 2).

As a follow-up to the FASTER trial, Dugoff *et al.* evaluated the effectiveness of the serum markers in identifying IUGR, defined either as below the tenth percentile or below the fifth percentile for birth weight [17,18]. Table 1 shows that PAPP-A, AFP, hCG, uE3 and inhibin identify a greater percentage of pregnancies with birth weight below the fifth percentile than those with birth weight below the tenth percentile. Since the below the tenth percentile group contains all of the pregnancies below the fifth percentile the difference between the two percentile cut-offs may not appear as significant. However, a comparison of those pregnancies with birth weight ≤5th percentile with those with birth weight between the sixth and tenth percentiles shows that the detection rates of the serum markers are significantly greater for the more extreme low birth weight group (Table 1).

**Table 1:** Detection rate (%) at a 5% false positive rate based on definition of IUGR using Birth Weight Percentiles.

Marker	Birth Weight Percentiles			*p-*Value *
	≤5th	≤10th	6th–10th	
PAPP-A	12.2	10.5	9.1	0.006
AFP	7.2	4.9	3.1	<0.001
hCG	11.9	10.7	9.7	0.06
uE3	2.7	2.2	1.8	0.104
Inhibin	13.1	10.5	8.5	<0.001

Data based on studies by Dugoff *et al.* [17,18]. * Comparison of 6th–10th percentile *vs.* ≤5th percentile (Fisher’s exact test). PAPP-A = pregnancy associated plasma protein-A, AFP = alpha-fetoprotein, hCG = human chorionic gonadotropin, uE3 = unconjugated estriol, Inhibin = Dimeric Inhibin A.

Spencer *et al.* [15] found that the pattern observed with the other serum markers was also true for second trimester free hCGβ. The authors found that the association of low second trimester free hCGβ (<0.5 MoM) was greater for birth weight below the third percentile (relative risk of 2.30) than for birth weight below the tenth percentile (relative risk of 1.98).

Roman *et al.* [76] used estimated fetal weight below the tenth percentile as a criterion for IUGR. In addition, the authors stratified the IUGR cases based on umbilical artery (UA) Doppler. Compared to pregnancies with normal growth, those pregnancies with IUGR and absent reverse end diastolic velocity (AREDV) had serum marker levels that were more significantly different than those pregnancies with IUGR and normal UA Doppler. In addition, using a combination of serum markers, 73% of the IUGR/AREDV cases were detected at a 5% false positive rate. By including maternal factors (history of chronic hypertension, lupus, pregestational diabetes and thrombophilia) in addition to serum markers 91% of IUGR/AREDV cases were identified. Further studies verifying the authors’ results could potentially lead to a direct screen for pregnancies at risk for IUGR with abnormal UA Doppler, a condition associated with significant morbidity, mortality and NICU admission.

## 4. Preterm Birth

In 2012, 9.9% of singleton births were preterm (<37 weeks) including 2.8% which were early preterm (<34 weeks) [77]. Identification of pregnancies at high risk for preterm birth based on short cervix can identify approximately one third of preterm births [43]. Recent randomized control trials have indicated that treatment with progesterone [41,42,43] or cervical cerclage [44] can significantly reduce preterm birth. Since only one third of early preterm births (<34 weeks) have short cervix below 1.5 cm the addition of biochemical and other biophysicial markers may lead to reduction in incidence of preterm birth and by extension a reduction in perinatal morbidity and mortality.

Several studies have evaluated the association of PAPP-A with preterm birth. Table 2 shows that PAPP-A is more strongly associated with early preterm birth than with preterm birth. At a 5% false positive rate, early preterm birth was identified in 9%–15% of cases compared to 5%–9% in preterm birth [16,17,53,54,78]. The positive likelihood ratio of PAPP-A below the fifth percentile ranged from 2 to 3 for early preterm birth. Goetzinger [79], evaluated PAPP-A at a 10% false positive rate and contrary to the other studies actually had higher detection in the preterm birth group (24%) compared to the early preterm birth group (20%). However, when maternal characteristics (African American race, Body Mass Index, Prior preterm birth, history of chronic hypertension, history of pre-gestational diabetes) were factored in, the detection rate was the same (38%) in both groups.

**Table 2:** Performance of PAPP-A in predicting early pre-term and pre-term delivery.

Author	Early Preterm				Preterm			
	GA at Delivery	DR (%) *	LR+	LR−	GA at Delivery	DR (%) *	LR+	LR−
Ong *et al.* [54]	<34	14.9	2.98	0.90	34–36	5.5	1.10	0.99
Smith *et al.* [53]	<32	14.0	2.80	0.91	32–36	9.5	1.90	0.95
Krantz *et al.* [16]	<34	9.4	1.88	0.95	-	-	-	-
Dugoff *et al.* [17]	<33	9.5	1.90	0.95	33–36	8.4	1.68	0.96
Spencer *et al.* [78]	<34	12.4	2.48	0.92	34–36	9.2	1.84	0.96
Spencer *et al.* [78]	<32	15.0	3.00	0.89	32–36	9.6	1.92	0.95
Goetzinger *et al.* [79]	<34	20	2.00	0.89	34–36	24	2.40	0.84
Goetzinger *et al.* [79] *^,†^	<34	38	3.80	0.69	34–36	38	3.80	0.69

GA = Gestational Age, DR = Detection Rate, LR = Likelihood Ratio. Preterm = delivery prior to 37 weeks, early preterm = delivery prior to 34 weeks. * All data at 5% false positive rate, except for Goetzinger *et al.* [79] which is at 10% false positive rate. ^†^ Includes maternal characteristics of African American race, body mass index, prior preterm birth, history of chronic hypertension and history of pre-gestational diabetes.

Dugoff *et al.* [18] evaluated the association of second trimester markers with early preterm birth ≤32 weeks. The detection (false positive rates) for AFP, hCG, uE3 and inhibin were 9% (5%), 11% (1.7%), 17% (6.0%), and 22% (3.1%). The combination of any two abnormal analytes had a sensitivity of 16% with a false positive rate of 2.9%, a positive likelihood ratio of 5.5 and a negative likelihood ratio (0 or 1 abnormal markers) of 0.87. The combination of both an elevated AFP and inhibin had the largest association with early preterm birth (Odds ratio = 20.37).

Data on the incidence of early preterm birth and short cervix can also be converted to likelihood ratios. Using the summarized data from Werner *et al.* [80], the likelihood ratio for birth before 34 weeks for cervix length <1.5 cm, 1.6–2.5 cm and >2.5 cm are 24.3, 2.5 and 0.9, respectively. In a separate study, Heath *et al.* estimated the likelihood ratio for birth before 32 weeks for cervix length <1 cm, 1–2 cm, 2–3 cm, 3–4 cm, 4–5 cm, 5–6 cm and 6–7 cm to be 51.52, 2.66, 0.71, 0.48, 0.24, 0.04 and 0.01, respectively [81]. These results are largely in agreement with those of Werner *et al.* [80]. Iams *et al.* determined that the risk of preterm birth in pregnancies with negative fibronectin, large cervical length and no history of preterm birth is 1% compared to 64% in pregnancies with positive fibronectin, small cervical length and history of preterm birth [82,83]. Although not currently routine practice, the data in these studies can be adjusted by incorporation of early biochemical marker likelihood ratios to further refine risks and improve detection of early preterm birth.

## 5. Fetal Loss

First trimester screening typically takes place beginning at 11 weeks. However, in some programs the blood sample for biochemistry testing is drawn prior to the ultrasound and may be collected as early as 9 weeks. As a result, data on fetal loss can be stratified into 3 timeframes; loss prior to nuchal translucency (NT) ultrasound, loss prior to 24 weeks gestation and loss after 24 weeks gestation.

Cuckle *et al.* [84] and Krantz *et al.* [85] evaluated the association of free hCGβ and PAPP-A with fetal viability at the time of the nuchal translucency exam. Cuckle *et al.* [84] examined 155 patients where blood was drawn prior to the NT examination and found nine patients with non-viable pregnancies at the time of the ultrasound. The medians for PAPP-A and free hCGβ in the 9 non-viable pregnancies were 0.25 and 0.78 respectively. Krantz *et al.* [85] found median MoMs of 0.47 and 0.42, respectively in 55 patients experiencing fetal loss prior to the NT ultrasound compared to a control group of 6464 unaffected patients. Using the predicted odds from logistic regression, the detection rate for a 1%, 3%, 5% and 10% false positive rate was 29%, 44%, 49% and 60% respectively [85].

A number of studies [17,62,86,87,88] have found that first trimester free hCGβ and PAPP-A are both associated with increased risk of early fetal loss between the time of the nuchal translucency exam and 24 weeks gestation. Dugoff *et al.* [87] evaluated a multiple marker approach to identify pregnancies at high risk for early fetal loss using a methodology similar to that used in calculating Down’s risk based on multivariate Gaussian distributions. The authors found that at a 5% false positive rate PAPP-A plus maternal characteristics (maternal age, body mass index, race, parity, previous loss prior to 24 weeks, preterm birth <37 weeks and threatened abortion) could detect 23% of early fetal loss. Including AFP and uE3 in the model resulted in a detection rate of 39%. Increasing the false positive rate to 10% resulted in a detection rate of 46%. Huang *et al.* [62] also evaluated a multiple marker approach to identify pregnancies at high risk for early fetal loss. In their model the combination of AFP, second trimester hCG and uE3 detected 42% of early fetal loss. Separately, Benn *et al.* [89] found that among patients with elevated AFP and simultaneously low uE3 (<0.7 MoM), there was a 9 fold increase in risk of fetal death.

The performance of screening for late fetal loss does not appear to be as effective with observed detection rates between 3%–20% at a 5% false positive rate [87,88,90]. The most promising approach was observed by Dugoff *et al.* [87], who reported that the combination of inhibin with maternal factors (body mass index and race) could lead to the detection of 20% and 29% of late fetal loss at a false positive rate of 5% and 10%, respectively.

Table 3 summarizes the performance of the various markers and their association with fetal loss prior to the nuchal translucency (NT) examination, between the NT examination and 24 weeks and after 24 weeks sorted by detection rate.

## 6. Placenta Accreta

Placenta accreta is a life-threatening obstetric complication resulting from abnormal placental implantation. The risk of placenta accreta increases significantly with placenta previa and the number of previous cesarean deliveries [91]. Currently, ultrasound and MRI are the best tools to diagnose placenta accreta although they have generally been applied only to high-risk pregnancies [92]. Therefore, improving the ability to identify pregnancies specifically at high risk for placenta accreta with biomarkers could help improve the efficient use of these imaging tools [92,93,94,95].

**Table 3:** Performance of screening marker protocols in identifying fetal loss.

Study	Protocol	Unaffected (*n*)	Cases (*n*)	DR (%) @ 5% FPR	LR+	LR−
**Loss Prior to NT**						
Krantz *et al.* [85]	PAPP-A	6464	55	36	7.1	0.68
Krantz *et al.* [85]	Free hCGβ	6464	55	47	9.5	0.56
Krantz *et al.* [85]	Free hCGβ + PAPP-A	6464	55	49	9.8	0.54
**Early Fetal Loss**						
Goetzl *et al.* [86]	PAPP-A	7932	75	12	2.4	0.93
Dugoff *et al.* [87]	PAPP-A	33,395	389	12	2.4	0.93
Spencer *et al.* [88]	Free hCGβ	47,770	230	12	2.4	0.93
Spencer *et al.* [88]	PAPP-A	47,770	230	15	3.0	0.89
Goetzl *et al.* [86]	Free hCGβ	7932	75	17	3.4	0.87
Dugoff *et al.* [87]	PAPP-A + Characteristics ^†^	32,631	194	23	4.6	0.81
Dugoff *et al.* [87]	uE3	32,631	194	24	4.8	0.80
Dugoff *et al.* [87]	AFP	32,631	194	29	5.8	0.75
Dugoff *et al.* [87]	uE3 + Characteristics ^†^	32,631	194	32	6.4	0.72
Dugoff *et al.* [87]	AFP + uE3 + PAPP-A	32,631	194	35	7.0	0.68
Dugoff *et al.* [87]	AFP + Characteristics ^†^	32,631	194	36	7.2	0.67
Dugoff *et al.* [87]	AFP + uE3 + PAPP-A + Characteristics ^†^	32,631	194	39	7.8	0.64
Huang *et al.* [62]	AFP + uE3 + hCG	141,698	296	42	8.4	0.61
**Late Fetal Loss**						
Goetzl *et al.* [86]	PAPP-A	7932	75	3	0.6	1.02
Goetzl *et al.* [86]	Free hCGβ	7932	75	7	1.4	0.98
Spencer *et al.* [88]	PAPP-A	47,770	225	9	1.8	0.96
Dugoff *et al.* [87]	PAPP-A	33,395	389	11	2.2	0.94
Spencer *et al.* [88]	Free hCGβ	47,770	225	12	2.4	0.93
Smith *et al.* [90]	Free hCGβ	8817	22	14	2.8	0.91
Dugoff *et al.* [87]	Inhibin	32,631	194	17	3.4	0.87
Smith *et al.* [90]	PAPP-A	8817	22	18	3.6	0.86
Dugoff *et al.* [87]	Inhibin + Characteristics ^‡^	32,631	194	20	4.0	0.84

DR = Detection Rate, FPR = False Positive Rate, LR+ = Positive Likelihood Ratio, LR− = Negative Likelihood Ratio, PAPP-A = pregnancy associated plasma protein A, hCG = human chorionic gonadotropin, AFP = alpha-fetoprotein, uE3 = unconjugated estriol, Inhibin = dimeric inhibin A. ^†^ Characteristics include maternal age, body mass index, race, parity, previous loss prior to 24 weeks, preterm birth <37 weeks and threatened abortion; ^‡^ Characteristics include body mass index and race.

Desai *et al.* found that high levels of PAPP-A were associated with increased risk of placenta accreta [49] and moreover that PAPP-A is not associated with placenta previa or previous cesarean. Similarly, Dugoff *et al.* [17] also showed that PAPP-A was not associated with previa. This indicates that PAPP-A could potentially be combined with the clinical evaluation of placenta previa and history of cesarean section to help identify those patients that are at high risk for placenta accreta. Using a continuous model, Desai *et al.* [49] found that a PAPP-A of 2 MoM was associated with a 2 fold increase in risk and that a PAPP-A of 3 MoM was associated with a 4-fold increase in risk. On the other-hand, a PAPP-A of 0.5 was associated with a 5 fold decrease in risk. These risk changes are equivalent to the change in risk of one or two additional cesarean deliveries or two fewer cesarean deliveries, respectively.

In the second trimester, Zelop *et al.* [50] and Kupfernic *et al.* [51] showed in small studies that elevated AFP (>2.5 MoM) was associated with accreta. Hung *et al.* [96] and Dreux *et al.* [97] found that second trimester levels of AFP and free hCGβ were elevated in accreta. When AFP was beyond 2.5 MoM the odds ratio for placenta accreta was 8–10. When free hCGβ was beyond 2.5 MoM the odds ratio was 4–8.

Further study into the development of algorithms encompassing multiple marker cross-trimester protocols, repeat marker testing, prior history of cesarean section and existence of previa are warranted.

## 7. Open Neural Tube Defects

The concept of prenatal screening began with the use of AFP for the detection of open neural tube defects (ONTDs) and evolved so that the main focus of serum marker screening is now chromosomal abnormalities [1,2,3,4,5,6,7,8,9,10,11]. Current advances in non-invasive cffDNA testing are not directed at the identification of pregnancies affected by ONTDs. A first trimester ultrasound is effective in identifying anencephaly [98] but less so in identifying open spina bifida [99] although newer techniques may improve detection [100].

A second trimester anatomy scan can be effective in identifying neural tube defects in specialized centers focused on high risk pregnancies [101]; however, it has been demonstrated to be less effective in general practice focused on low risk pregnancies [102]. Therefore, ACOG recommends that maternal serum AFP screening be offered to all pregnant women and that those found to be at high risk for ONTD may be offered specialized ultrasound examination to identify the defect [103]. The importance of prenatal screening and detection of such defects may be even more relevant now that fetal surgery offers the promise of improved outcome in certain cases of open spina bifida [46,47,48].

The serum screen for open neural tube defects is straightforward with labs using either a 2.0 MoM or 2.5 MoM cut-off. The detection rate of open spina bifida is approximately 10 percentage points greater with a 2 MoM rather than a 2.5 MoM cutoff [104,105]. In addition, there have been significant improvements in AFP assays since screening was first introduced with radioimmunoassay in the 1970s. As a result the distribution of AFP is much narrower and thus the use of a 2.0 MoM can result in false positive rates of 2% or less [105]. Even though most laboratories report a patient-specific risk for open spina bifida the reported risk values may not be as accurate because many of the a priori risks incorporated into algorithms are based on incidence data collected prior to the implementation of folic acid dietary supplementation which has significantly reduced the risk of neural tube defects [106].

## 8. Conclusions

For adverse outcomes such as IUGR, preeclampsia and preterm birth the clinical presentation may vary widely with respect to maternal/fetal morbidity and mortality. The more severe form of these adverse outcomes have significantly higher rates of severe morbidity and mortality. The information presented in this review indicates that there is improved performance of serum markers with respect to the more severe form of various pregnancy complications. Moreover, the most severe cases tend to occur less frequently than the milder forms of these conditions (Table 4). As a result, studies which evaluate the association of biomarkers with a broad definition of a given condition may underestimate the ability of such markers to identify pregnancies that are destined to develop the more severe form of the condition. Therefore, more effort should be made to narrowly define specific adverse outcomes which may be identified by maternal serum markers. Using these narrowly defined outcomes, clinicians can decide whether screening is worthwhile based on incidence rates and clinical impact.

**Table 4:** Inverse relationship between incidence of the subcategory of adverse outcome and rate of severe morbidity and mortality associated with the subcategory.

	Preeclampsia		Preterm Birth		IUGR	
Description	Early Onset <34 Weeks	Late Onset ≥34 Weeks	<34 Weeks	34–36 Weeks	Abnormal UA	Normal UA
Incidence	0.4%	2.7%	2.1%	8.2%	3.1%	6.3%
Rate of Severe Morbidity and Mortality	25%	2.5%	10%	2.6%	13%	1.4%

UA = Umbilical Artery Doppler, IUGR = Intrauterine growth restriction. Each line represents an adverse outcome. Results based on published data for preeclampsia [35], preterm birth [80,107,108] and IUGR [70].

Published performance data among different studies are often inconsistent due to discrepancies in a number of factors such as the definition and/or description of the severity of the condition, the marker cutoffs used and the maternal characteristics incorporated into risk algorithms. Optimally, a risk-based approach similar to that used in aneuploidy screening would be used for each disease state, in which consistent definition of the disease state, continuous multiple marker likelihood estimates and consistent estimates of a priori risks based on maternal characteristics were incorporated. Additionally, refinements to the risk based on follow-up assessments after the completion of serum screening could further improve the process.

Clinicians are faced with a difficult dilemma in which they must balance the potential benefits of non-invasive genetic screening while not losing sight of the potential pitfalls in missing other adverse outcomes especially since there now appears to be opportunity to improve those outcomes with effective treatments. Aspirin shows great promise if administered prior to 16 weeks in reducing the risk of preeclampsia, IUGR, preterm birth and fetal death. Some of the protocols described above include second trimester markers and would require completion by 16 weeks to maximize the benefits of aspirin administration. However, it is likely that the effectiveness of aspirin is not based on a simple dichotomy of <16 weeks and ≥16 weeks so aspirin may still be effective at 17–18 weeks even if less so than at 16 weeks. More research is needed to evaluate the association between effectiveness and time of initiation of aspirin treatment.

Moving forward, the goal should be to develop and implement high-performance direct screening protocols for specifically defined adverse outcomes. When evaluating the adoption of cffDNA testing for aneuploidy, clinicians should ensure that they continue to utilize existing screening protocols or new direct screens to identify pregnancies at risk for adverse outcomes. Otherwise, there may potentially be an increase in the overall morbidity and mortality in the population.

## References

[1] Macri J.N., Weiss R.R. (1982). Prenatal serum alpha-fetoprotein screening for neural tube defects. Obstet. Gynecol..

[2] UK Collaborative Study (1982). Estimating an individual’s risk of having a fetus with open spina bifida and the value of repeat alpha-fetoprotein testing. Fourth report of the UK collaborative study on alpha-fetoprotein in relation to neural tube defects. J. Epidemiol. Community Health.

[3] Krantz D.A., Larsen J.W., Buchanan P.D., Macri J.N. (1996). First-trimester Down syndrome screening: Free beta-human chorionic gonadotropin and pregnancy-associated plasma protein A. Am. J. Obstet. Gynecol..

[4] Snijders R.J., Noble P., Sebire N., Souka A., Nicolaides K.H. (1998). UK multicentre project on assessment of risk of trisomy 21 by maternal age and fetal nuchal-translucency thickness at 10–14 weeks of gestation. Fetal Medicine Foundation First Trimester Screening Group. Lancet.

[5] Orlandi F., Damiani G., Hallahan T.W., Krantz D.A., Macri J.N. (1997). First-trimester screening for fetal aneuploidy: Biochemistry and nuchal translucency. Ultrasound Obstet. Gynecol..

[6] Krantz D.A., Hallahan T.W., Orlandi F., Buchanan P., Larsen J.W., Macri J.N. (2000). First-trimester Down syndrome screening using dried blood biochemistry and nuchal translucency. Obstet. Gynecol..

[7] Wapner R., Thom E., Simpson J.L., Pergament E., Silver R., Filkins K., Platt L., Mahoney M., Johnson A., Hogge W.A. (2003). First-trimester screening for trisomies 21 and 18. N. Engl. J. Med..

[8] Malone F.D., Canick J.A., Ball R.H., Nyberg D.A., Comstock C.H., Bukowski R., Berkowitz R.L., Gross S.J., Dugoff L., Craigo S.D. (2005). First-trimester or second-trimester screening, or both, for Down’s syndrome. N. Engl. J. Med..

[9] Spencer K., Nicolaides K.H. (2002). A first trimester trisomy 13/trisomy 18 risk algorithm combining fetal nuchal translucency thickness, maternal serum free beta-hCG and PAPP-A. Prenat. Diagn..

[10] Cuckle H., Benn P., Wright D. (2005). Down syndrome screening in the first and/or second trimester: Model predicted performance using meta-analysis parameters. Semin. Perinatol..

[11] Cicero S., Curcio P., Papageorghiou A., Sonek J., Nicolaides K. (2001). Absence of nasal bone in fetuses with trisomy 21 at 11–14 weeks of gestation: An observational study. Lancet.

[12] Davenport D.M., Macri J.N. (1983). The clinical significance of low maternal serum alpha-fetoprotein. Am. J. Obstet. Gynecol..

[13] Katz V.L., Chescheir N.C., Cefalo R.C. (1990). Unexplained elevations of maternal serum alpha-fetoprotein. Obstet. Gynecol. Surv..

[14] Gross S.J., Phillips O.P., Shulman L.P., Bright N.L., Dungan J.S., Simpson J.L., Elias S. (1994). Adverse perinatal outcome in patients screen-positive for neural tube defects and fetal Down syndrome. Prenat. Diagn..

[15] Spencer K. (2000). Second-trimester prenatal screening for Down syndrome and the relationship of maternal serum biochemical markers to pregnancy complications with adverse outcome. Prenat. Diagn..

[16] Krantz D., Goetzl L., Simpson J.L., Thom E., Zachary J., Hallahan T.W., Silver R., Pergament E., Platt L.D., Filkins K. (2004). Association of extreme first-trimester free human chorionic gonadotropin-beta, pregnancy-associated plasma protein A, and nuchal translucency with intrauterine growth restriction and other adverse pregnancy outcomes. Am. J. Obstet. Gynecol..

[17] Dugoff L., Hobbins J.C., Malone F.D., Porter T.F., Luthy D., Comstock C.H., Hankins G., Berkowitz R.L., Merkatz I., Craigo S.D. (2004). First-trimester maternal serum PAPP-A and free-beta subunit human chorionic gonadotropin concentrations and nuchal translucency are associated with obstetric complications: A population-based screening study (the FASTER Trial). Am. J. Obstet. Gynecol..

[18] Dugoff L., Hobbins J.C., Malone F.D., Vidaver J., Sullivan L., Canick J.A., Lambert-Messerlian G.M., Porter T.F., Luthy D.A., Comstock C.H. (2005). Quad screen as a predictor of adverse pregnancy outcome. Obstet. Gynecol..

[19] Dugoff L., Society for Maternal-Fetal Medicine (2010). First- and second-trimester maternal serum markers for aneuploidy and adverse obstetric outcomes. Obstet. Gynecol..

[20] Kuc S., Wortelboer E.J., van Rijn B.B., Franx A., Visser G.H., Schielen P.C. (2011). Evaluation of 7 serum biomarkers and uterine artery Doppler ultrasound for first-trimester prediction of preeclampsia: A systematic review. Obstet. Gynecol. Surv..

[21] Goetzl L. (2010). Adverse pregnancy outcomes after abnormal first-trimester screening for aneuploidy. Clin. Lab. Med..

[22] Gagnon A., Wilson R.D., Audibert F., Allen V.M., Blight C., Brock J.A., Désilets V.A., Johnson J.A., Langlois S., Summers A. (2008). Obstetrical complications associated with abnormal maternal serum markers analytes. J. Obstet. Gynaecol. Can..

[23] Morris R.K., Cnossen J.S., Langejans M., Robson S.C., Kleijnen J., Ter Riet G., Mol B.W., van der Post J.A., Khan K.S. (2008). Serum screening with Down’s syndrome markers to predict pre-eclampsia and small for gestational age: Systematic review and meta-analysis. BMC Pregnancy Childbirth.

[24] Goetzinger K.R., Odibo A.O. (2014). Screening for abnormal placentation and adverse pregnancy outcomes with maternal serum biomarkers in the second trimester. Prenat. Diagn..

[25] Lo Y.M., Corbetta N., Chamberlain P.F., Rai V., Sargent I.L., Redman C.W., Wainscoat J.S. (1997). Presence of fetal DNA in maternal plasma and serum. Lancet.

[26] Ehrich M., Deciu C., Zwiefelhofer T., Tynan J.A., Cagasan L., Tim R., Lu V., McCullough R., McCarthy E., Nygren A.O. (2011). Noninvasive detection of fetal trisomy 21 by sequencing of DNA in maternal blood: A study in a clinical setting. Am. J. Obstet. Gynecol..

[27] Palomaki G.E., Kloza E.M., Lambert-Messerlian G.M., Haddow J.E., Neveux L.M., Ehrich M., van den Boom D., Bombard A.T., Deciu C., Grody W.W. (2011). DNA sequencing of maternal plasma to detect Down syndrome: An international clinical validation study. Genet. Med..

[28] Bianchi D.W., Platt L.D., Goldberg J.D., Abuhamad A.Z., Sehnert A.J., Rava R.P., MatErnal BLood IS Source to Accurately diagnose fetal aneuploidy (MELISSA) Study Group (2012). Genome-wide fetal aneuploidy detection by maternal plasma DNA sequencing. Obstet. Gynecol..

[29] Palomaki G.E., Deciu C., Kloza E.M., Lambert-Messerlian G.M., Haddow J.E., Neveux L.M., Ehrich M., van den Boom D., Bombard A.T., Grody W.W. (2012). DNA sequencing of maternal plasma reliably identifies trisomy 18 and trisomy 13 as well as Down syndrome: An international collaborative study. Genet. Med..

[30] Srinivasan A., Bianchi D.W., Huang H., Sehnert A.J., Rava R.P. (2013). Noninvasive detection of fetal subchromosome abnormalities via deep sequencing of maternal plasma. Am. J. Hum. Genet..

[31] American College of Obstetricians and Gynecologists Committee on Genetics (2012). Committee Opinion No. 545: Noninvasive prenatal testing for fetal aneuploidy. Obstet. Gynecol..

[32] Cuckle H., Benn P., Pergament E. (2013). Maternal cfDNA screening for Down syndrome—A cost sensitivity analysis. Prenat. Diagn..

[33] Petersen O., Vogel I., Ekelund C., Hyett J., Tabor A. (2014). Potential diagnostic consequences of applying non-invasive prenatal testing (NIPT); a population-based study from a country with existing first trimester screening. Ultrasound Obstet. Gynecol..

[34] Poon L.C., Musci T., Song K., Syngelaki A., Nicolaides K.H. (2013). Maternal plasma cell-free fetal and maternal DNA at 11–13 weeks’ gestation: Relation to fetal and maternal characteristics and pregnancy outcomes. Fetal Diagn. Ther..

[35] Lisonkova S., Joseph K.S. (2013). Incidence of preeclampsia: Risk factors and outcomes associated with early- *versus* late-onset disease. Am. J. Obstet. Gynecol..

[36] Wapner R.J., Martin C.L., Levy B., Ballif B.C., Eng C.M., Zachary J.M., Savage M., Platt L.D., Saltzman D., Grobman W.A. (2012). Chromosomal microarray *versus* karyotyping for prenatal diagnosis. N. Engl. J. Med..

[37] Askie L.M., Duley L., Henderson-Smart D.J., Stewart L.A. (2007). Antiplatelet agents for prevention of pre-eclampsia: A meta-analysis of individual patient data. Lancet.

[38] Bujold E., Roberge S., Lacasse Y., Bureau M., Audibert F., Marcoux S., Forest J.C., Giguère Y. (2010). Prevention of preeclampsia and intrauterine growth restriction with aspirin started in early pregnancy: A meta-analysis. Obstet. Gynecol..

[39] Roberge S., Villa P., Nicolaides K., Giguère Y., Vainio M., Bakthi A., Ebrashy A., Bujold E. (2012). Early administration of low-dose aspirin for the prevention of preterm and term preeclampsia: A systematic review and meta-analysis. Fetal Diagn. Ther..

[40] Roberge S., Nicolaides K.H., Demers S., Villa P., Bujold E. (2013). Prevention of perinatal death and adverse perinatal outcome using low-dose aspirin: A meta-analysis. Ultrasound Obstet. Gynecol..

[41] Meis P.J., Klebanoff M., Thom E., Dombrowski M.P., Sibai B., Moawad A.H., Spong C.Y., Hauth J.C., Miodovnik M., Varner M.W. (2003). Prevention of recurrent preterm delivery by 17 alpha-ydroxyprogesterone caproate. N. Engl. J. Med..

[42] Da Fonseca E.B., Bittar R.E., Carvalho M.H., Zugaib M. (2003). Prophylactic administration of progesterone by vaginal suppository to reduce the incidence of spontaneous preterm birth in women at increased risk: A randomized placebo-controlled double-blind study. Am. J. Obstet. Gynecol..

[43] Fonseca E.B., Celik E., Parra M., Singh M., Nicolaides K.H., Fetal Medicine Foundation Second Trimester Screening Group (2007). Progesterone and the risk of preterm birth among women with a short cervix. N. Engl. J. Med..

[44] Owen J., Hankins G., Iams J.D., Berghella V., Sheffield J.S., Perez-Delboy A., Egerman R.S., Wing D.A., Tomlinson M., Silver R. (2009). Multicenter randomized trial of cerclage for preterm birth prevention in high-risk women with shortened midtrimester cervical length. Am. J. Obstet. Gynecol..

[45] Berghella V., Rafael T.J., Szychowski J.M., Rust O.A., Owen J. (2011). Cerclage for short cervix on ultrasonography in women with singleton gestations and previous preterm birth: A meta-analysis. Obstet. Gynecol..

[46] Bruner J.P., Tulipan N., Paschall R.L., Boehm F.H., Walsh W.F., Silva S.R., Hernanz-Schulman M., Lowe L.H., Reed G.W. (1999). Fetal surgery for myelomeningocele and the incidence of shunt-dependent hydrocephalus. JAMA.

[47] Sutton L.N., Adzick N.S., Bilaniuk L.T., Johnson M.P., Crombleholme T.M., Flake A.W. (1999). Improvement in hindbrain herniation demonstrated by serial fetal magnetic resonance imaging following fetal surgery for myelomeningocele. JAMA.

[48] Adzick N.S., Thom E.A., Spong C.Y., Brock J.W., Burrows P.K., Johnson M.P., Howell L.J., Farrell J.A., Dabrowiak M.E., Sutton L.N. (2011). A randomized trial of prenatal *versus* postnatal repair of myelomeningocele. N. Engl. J. Med..

[49] Desai N., Krantz D., Roman A., Fleischer A., Boulis S., Rochelson B. (2014). Elevated first trimester PAPP-A is associated with increased risk of placenta accreta. Prenat. Diagn..

[50] Zelop C., Nadel A., Frigoletto F.D., Pauker S., MacMillan M., Benacerraf B.R. (1992). Placenta accreta/percreta/increta: A cause of elevated maternal serum alpha-fetoprotein. Obstet. Gynecol..

[51] Kupferminc M.J., Tamura R.K., Wigton T.R., Glassenberg R., Socol M.L. (1993). Placenta accreta is associated with elevated maternal serum alpha-fetoprotein. Obstet. Gynecol..

[52] Yaron Y., Heifetz S., Ochshorn Y., Lehavi O., Orr-Urtreger A. (2002). Decreased first trimester PAPP-A is a predictor of adverse pregnancy outcome. Prenat. Diagn..

[53] Smith G.C., Stenhouse E.J., Crossley J.A., Aitken D.A., Cameron A.D., Connor J.M. (2002). Early pregnancy levels of pregnancy-associated plasma protein A and the risk of intrauterine growth restriction, premature birth, pre eclampsia, and stillbirth. J. Clin. Endocrinol. Metab..

[54] Ong C.Y., Liao A.W., Spencer K., Munim S., Nicolaides K.H. (2000). First trimester maternal serum free beta human chorionic gonadotrophin and pregnancy associated plasma protein A as predictors of pregnancy complications. Br. J. Obstet. Gynaecol..

[55] Goetzinger K.R., Singla A., Gerkowicz S., Dicke J.M., Gray D.L., Odibo A.O. (2010). Predicting the risk of pre-eclampsia between 11 and 13 weeks’ gestation by combining maternal characteristics and serum analytes, PAPP-A and free β-hCG. Prenat. Diagn..

[56] Spencer K., Cowans N.J., Nicolaides K.H. (2008). Low levels of maternal serum PAPP-A in the first trimester and the risk of pre-eclampsia. Prenat. Diagn..

[57] Kang J.H., Farina A., Park J.H., Kim S.H., Kim J.Y., Rizzo N., Elmakky A., Jun H.S., Hahn W.B., Cha D.H. (2008). Down syndrome biochemical markers and screening for preeclampsia at first and second trimester: Correlation with the week of onset and the severity. Prenat. Diagn..

[58] Olsen R.N., Woelkers D., Dunsmoor-Su R., Lacoursiere D.Y. (2012). Abnormal second-trimester serum analytes are more predictive of preterm preeclampsia. Am. J. Obstet. Gynecol..

[59] Akolekar R., Syngelaki A., Sarquis R., Zvanca M., Nicolaides K.H. (2011). Prediction of early, intermediate and late pre-eclampsia from maternal factors, biophysical and biochemical markers at 11–13 weeks. Prenat. Diagn..

[60] D’Antonio F., Rijo C., Thilaganathan B., Akolekar R., Khalil A., Papageourgiou A., Bhide A. (2013). Association between first-trimester maternal serum pregnancy-associated plasma protein-A and obstetric complications. Prenat. Diagn..

[61] Kuc S., Koster M.P., Franx A., Schielen P.C., Visser G.H. (2013). Maternal characteristics, mean arterial pressure and serum markers in early prediction of preeclampsia. PLoS One.

[62] Huang T., Hoffman B., Meschino W., Kingdom J., Okun N. (2010). Prediction of adverse pregnancy outcomes by combinations of first and second trimester biochemistry markers used in the routine prenatal screening of Down syndrome. Prenat. Diagn..

[63] Akolekar R., Syngelaki A., Poon L., Wright D., Nicolaides K.H. (2013). Competing risks model in early screening for preeclampsia by biophysical and biochemical markers. Fetal Diagn. Ther..

[64] Poon L.C., Syngelaki A., Akolekar R., Lai J., Nicolaides K.H. (2013). Combined screening for preeclampsia and small for gestational age at 11–13 weeks. Fetal Diagn Ther..

[65] Cuckle H.S. (2011). Screening for pre-eclampsia—Lessons from aneuploidy screening. Placenta.

[66] Kagan K.O., Hoopmann M., Abele H., Alkier R., Lüthgens K. (2012). First-trimester combined screening for trisomy 21 with different combinations of placental growth factor, free β-human chorionic gonadotropin and pregnancy-associated plasma protein-A. Ultrasound Obstet. Gynecol..

[67] Donalson K., Turner S., Morrison L., Liitti P., Nilsson C., Cuckle H. (2013). Maternal serum placental growth factor and α-fetoprotein testing in first trimester screening for Down syndrome. Prenat. Diagn..

[68] Johnson J., Pastuck M., Metcalfe A., Connors G., Krause R., Wilson D., Cuckle H. (2013). First-trimester Down syndrome screening using additional serum markers with and without nuchal translucency and cell-free DNA. Prenat. Diagn..

[69] Lausman A., Mccarthy F.P., Walker M., Kingdom J. (2012). Screening, diagnosis, and management of intrauterine growth restriction. J. Obstet. Gynaecol. Can..

[70] Unterscheider J., Daly S., Geary M.P., Kennelly M.M., McAuliffe F.M., O’Donoghue K., Hunter A., Morrison J.J., Burke G., Dicker P. (2013). Optimizing the definition of intrauterine growth restriction: The multicenter prospective PORTO Study. Am. J. Obstet. Gynecol..

[71] Pilalis A., Souka A.P., Antsaklis P., Daskalakis G., Papantoniou N., Mesogitis S., Antsaklis A. (2007). Screening for pre-eclampsia and fetal growth restriction by uterine artery Doppler and PAPP-A at 11–14 weeks’ gestation. Ultrasound Obstet. Gynecol..

[72] Carbone J.F., Tuuli M.G., Bradshaw R., Liebsch J., Odibo A.O. (2012). Efficiency of first-trimester growth restriction and low pregnancy-associated plasma protein-A in predicting small for gestational age at delivery. Prenat. Diagn..

[73] Spencer K., Cowans N.J., Avgidou K., Molina F., Nicolaides K.H. (2008). First-trimester biochemical markers of aneuploidy and the prediction of small-for-gestational age fetuses. Ultrasound Obstet. Gynecol..

[74] Kavak Z.N., Basgul A., Elter K., Uygur M., Gokaslan H. (2006). The efficacy of first-trimester PAPP-A and free beta hCG levels for predicting adverse pregnancy outcome. J. Perinat. Med..

[75] Leung T.Y., Sahota D.S., Chan L.W., Law L.W., Fung T.Y., Leung T.N., Lau T.K. (2008). Prediction of birth weight by fetal crown-rump length and maternal serum levels of pregnancy-associated plasma protein-A in the first trimester. Ultrasound Obstet. Gynecol..

[76] Roman A., Desai N., Krantz D., Liu H.P., Rosner J., Vohra N., Rochelson B. (2014). Maternal serum analytes as predictors of IUGR with different degrees of placental vascular dysfunction. Prenat. Diagn..

[77] Centers for Disease Control and Prevention, National Center for Health Statistics Gestational Length and Birthweight 2012. http://nchs/vitalstats.htm.

[78] Spencer K., Cowans N.J., Molina F., Kagan K.O., Nicolaides K.H. (2008). First-trimester ultrasound and biochemical markers of aneuploidy and the prediction of preterm or early preterm delivery. Ultrasound Obstet. Gynecol..

[79] Goetzinger K.R., Cahill A.G., Kemna J., Odibo L., Macones G.A., Odibo A.O. (2012). First-trimester prediction of preterm birth using ADAM12, PAPP-A, uterine artery Doppler, and maternal characteristics. Prenat. Diagn..

[80] Werner E.F., Han C.S., Pettker C.M., Buhimschi C.S., Copel J.A., Funai E.F., Thung S.F. (2011). Universal cervical-length screening to prevent preterm birth: A cost-effectiveness analysis. Ultrasound Obstet. Gynecol..

[81] Heath V.C.F., Southall T.R., Souka A.P., Elisseou A., Nicolaides K.H. (1998). Cervical length at 23 weeks of gestation: Prediction of spontaneous preterm delivery. Ultrasound Obstet. Gynecol..

[82] Iams J.D., Goldenberg R.L., Meis P.J., Mercer B.M., Moawad A., Das A., Thom E., McNellis D., Copper R.L., Johnson F. (1996). The length of the cervix and the risk of spontaneous premature delivery. National Institute of Child Health and Human Development Maternal Fetal Medicine Unit Network. N. Engl. J. Med..

[83] Iams J.D., Creasy R.K., Creasy R.K., Resnik R. (2004). Preterm labor and delivery. Maternal-Fetal Medicine Principals and Practice.

[84] Cuckle H.S., Sehmi I.K., Jones R.G., Mason G. (1999). Low maternal serum PAPP-A and fetal viability. Prenat. Diagn..

[85] Krantz D., Hallahan T., Ishack S., Macri V.J., Macri J.N. (2006). First-trimester maternal dried blood Down syndrome screening marker levels in early pregnancy loss. Prenat. Diagn..

[86] Goetzl L., Krantz D., Simpson J.L., Silver R.K., Zachary J.M., Pergament E., Platt L.D., Mahoney M.J., Wapner R.J. (2004). Pregnancy-associated plasma protein A, free beta-hCG, nuchal translucency, and risk of pregnancy loss. Obstet. Gynecol..

[87] Dugoff L., Cuckle H.S., Hobbins J.C., Malone F.D., Belfort M.A., Nyberg D.A., Comstock C.H., Saade G.R., Eddleman K.A., Dar P. (2008). Prediction of patient-specific risk for fetal loss using maternal characteristics and first- and second-trimester maternal serum Down syndrome markers. Am. J. Obstet. Gynecol..

[88] Spencer K., Cowans N.J., Avgidou K., Nicolaides K.H. (2006). First-trimester ultrasound and biochemical markers of aneuploidy and the prediction of impending fetal death. Ultrasound Obstet. Gynecol..

[89] Benn P.A., Craffey A., Horne D., Ramsdell L., Rodis J.F. (2000). Elevated maternal serum alpha-fetoprotein with low unconjugated estriol and the risk for lethal perinatal outcome. J. Matern. Fetal Med..

[90] Smith G.C.S., Crossley J.A., Aitken D.A., Pell J.P., Cameron A.D., Connor J.M., Dobbie R. (2004). First trimester placentation and the risk of antepartum stillbirth. JAMA.

[91] Silver R.M., Landon M.B., Rouse D.J., Leveno K.J., Spong C.Y., Thom E.A., Moawad A.H., Caritis S.N., Harper M., Wapner R.J. (2006). Maternal morbidity associated with multiple repeat cesarean deliveries. Obstet. Gynecol..

[92] Warshak C.R., Ramos G.A., Eskander R., Benirschke K., Saenz C.C., Kelly T.F., Moore T.R., Resnik R. (2010). Effect of predelivery diagnosis in 99 consecutive cases of placenta accreta. Obstet. Gynecol..

[93] Wright J.D., Herzog T.J., Shah M., Bonanno C., Lewin S.N., Cleary K., Simpson L.L., Gaddipati S., Sun X., D’Alton M.E. (2010). Regionalization of care for obstetric hemorrhage and its effect on maternal mortality. Obstet. Gynecol..

[94] Briery C.M., Rose C.H., Hudson W.T., Lutgendorf M.A., Magann E.F., Chauhan S.P., Morrison J.C. (2007). Planned *vs.* emergent cesarean hysterectomy. Am. J. Obstet. Gynecol..

[95] Eller A.G., Bennett M.A., Sharshiner M., Masheter C., Soisson A.P., Dodson M., Silver R.M. (2011). Maternal morbidity in cases of placenta accreta managed by a multidisciplinary care team compared with standard obstetric care. Obstet. Gynecol..

[96] Hung T.H., Shau W.Y., Hsieh C.C., Chiu T.H., Hsu J.J., Hsieh T.T. (1999). Risk factors for placenta accreta. Obstet. Gynecol..

[97] Dreux S., Salomon L.J., Muller F., Goffinet F., Oury J.F., Sentilhes L., ABA Study Group (2012). Second-trimester maternal serum markers and placenta accreta. Prenat. Diagn..

[98] Johnson S.P., Sebire N.J., Snijders R.J., Tunkel S., Nicolaides K.H. (1997). Ultrasound screening for anencephaly at 10–14 weeks of gestation. Ultrasound Obstet. Gynecol..

[99] Sebire N.J., Noble P.L., Thorpe-Beeston J.G., Snijders R.J., Nicolaides K.H. (1997). Presence of the “lemon” sign in fetuses with spina bifida at the 10–14-week scan. Ultrasound Obstet. Gynecol..

[100] Chaoui R., Benoit B., Heling K.S., Kagan K.O., Pietzsch V., Sarut Lopez A., Tekesin I., Karl K. (2011). Prospective detection of open spina bifida at 11–13 weeks by assessing intracranial translucency and posterior brain. Ultrasound Obstet. Gynecol..

[101] Lennon C.A., Gray D.L. (1999). Sensitivity and specificity of ultrasound for the detection of neural tube and ventral wall defects in a high-risk population. Obstet. Gynecol..

[102] Crane J.P., LeFevre M.L., Winborn R.C., Evans J.K., Ewigman B.G., Bain R.P., Frigoletto F.D., McNellis D. (1994). A randomized trial of prenatal ultrasonographic screening: Impact on the detection, management, and outcome of anomalous fetuses. The RADIUS Study Group. Am. J. Obstet. Gynecol..

[103] Cheschier N., ACOG Committee on Practice Bulletins-Obstetrics (2003). ACOG practice bulletin. Neural tube defects. Number 44, July 2003. Int. J. Gynaecol. Obstet..

[104] Milunsky A., Jick S.S., Bruell C.L., MacLaughlin D.S., Tsung Y.K., Jick H., Rothman K.J., Willett W. (1989). Predictive values, relative risks, and overall benefits of high and low maternal serum alpha-fetoprotein screening in singleton pregnancies: New epidemiologic data. Am. J. Obstet. Gynecol..

[105] Wald N.J., Hackshaw A.K., George L.M. (2000). Assay precision of serum alpha fetoprotein in antenatal screening for neural tube defects and Down’s syndrome. J. Med. Screen..

[106] Centers for Disease Control (CDC) (1991). Use of folic acid for prevention of spina bifida and other neural tube defects—1983–1991. MMWR Morb. Mortal. Wkly. Rep..

[107] Moster D., Lie R., Markestad T. (2008). Long-term medical and social consequences of preterm birth. N. Engl. J. Med..

[108] Clements K., Barfield W., Ayadi M., Wilber N. (2007). Preterm birth associated cost of early intervention services: An analysis by gestational age. Pediatrics.

